# Robotic partial nephrectomy is associated with a lower incidence of urine leakage following nephron-sparing surgery for kidney tumors compared to open and laparoscopic approaches

**DOI:** 10.1007/s00345-025-05651-z

**Published:** 2025-04-28

**Authors:** Husny Mahmud, Tomer Erlich, Dorit E. Zilberman, Barak Rosenzweig, Orith Portnoy, Zohar A. Dotan

**Affiliations:** 1 Department of Urology, Faculty of Medicine, Sheba Medical Center, Tel Aviv University, Tel Hashomer, Tel Aviv, Israel; 2 Diagnostic Imaging Department, Faculty of Medicine, Sheba Medical Center, Tel Aviv University, Tel Hashomer, Tel Aviv, Israel; 3 Department of Urology, The Chaim Sheba Medical Center, Tel Hashomer, Ramat Gan, 52621 Israel

**Keywords:** Partial nephrectomy, Urine leakage, Robotic-assisted partial nephrectomy, RENAL nephrometry, T1b renal tumors, Minimally invasive urology

## Abstract

**Purpose:**

Urine leakage (UL) is a recognized complication after partial nephrectomy (PN). This study aimed to determine the incidence of UL and identify key risk factors, including tumor size and surgical approach, to clarify the impact of robotic-assisted, laparoscopic, and open PN on postoperative outcomes.

**Methods:**

A retrospective review of 785 consecutive clinical T1 PN cases (2012–2022) was undertaken. UL was defined as biochemically confirmed uriniferous drain output ≥ 50 mL day-1 persisting ≥ 3 days. The overall incidence of UL was assessed, and multivariable statistical tests evaluated potential predictors of leakage. (19 events; EPV = 3.8; hypothesisgenerating).

**Results:**

Of the 785 patients, 50.7% had RAPN, 33.8% OPN, and 15.5% LPN. The overall incidence of UL was 2.4%. RAPN demonstrated the lowest leakage rate (0.75%), compared with OPN (3.7%) and LPN (4.91%) (*p* = 0.03), representing a five-fold reduction in UL risk compared to open and laparoscopic approaches. Patients with T1b tumors were significantly more prone to leakage than those with T1a tumors (15.8% vs. 0.99%; multivariable odds ratio (OR) = 18.8, 95% CI = 7.15–49.44; *p* < 0.0001). Longer operative and ischemia times were also associated with higher leakage risk. All UL cases resolved with conservative or minimally invasive interventions.

**Conclusions:**

Surgical approach, operative duration, ischemia time, and tumor size (T1b vs. T1a) emerged as principal predictors of postoperative UL. RAPN conferred a notably lower leakage risk compared to OPN and LPN, underscoring its advantages for nephron-sparing surgery, particularly in complex renal tumors requiring meticulous collecting-system closure.

**Trial registration:**

Not applicable (retrospective).

## Introduction

Partial nephrectomy (PN) has become the standard treatment for small renal masses (clinical T1), aiming to preserve renal parenchyma and mitigate the risk of chronic kidney disease [1–5]. While open partial nephrectomy (OPN) established early efficacy, the emergence of minimally invasive techniques—laparoscopic (LPN) and robotic-assisted partial nephrectomy (RAPN)—has further reduced morbidity and hospital stays [6, 7, 8]. RAPN, in particular, offers advantages such as enhanced visualization and suturing dexterity.

Despite these refinements, urinary leakage (UL) remains a notable postoperative complication, potentially leading to prolonged hospitalization, additional interventions, and in severe cases, compromised renal function [9, 10]. The low incidence of UL in many series (reportedly 0–33%) underscores the difficulty of adequately characterizing risk factors and drawing robust conclusions [10]. Moreover, prospective randomized comparisons of UL rates across open, laparoscopic, and robotic PN are lacking.

In light of these gaps, we performed a single-center retrospective study to (1) determine the incidence of UL following PN for T1 renal tumors, (2) identify clinical and perioperative risk factors (including tumor size and complexity), and (3) compare outcomes across OPN, LPN, and RAPN. We hypothesized that RAPN might confer a lower rate of UL owing to improved surgical ergonomics, but we also acknowledge that our sample’s low event rate may limit generalizability. Therefore, this study aims to guide patient counseling and surgical decision-making regarding nephron-sparing approaches.

## Materials and methods

### Study design and patient population

We performed a retrospective review of 785 patients with clinical T1 renal tumors who underwent PN at our institution between January 2012 and December 2022. Patients were included if they had a solitary renal tumor managed with either RAPN, LPN, or OPN. Patient demographics (age, gender, body mass index [BMI], American Society of Anesthesiologists [ASA] score, baseline estimated glomerular filtration rate [eGFR], and presence of chronic kidney disease [CKD]) were recorded. Tumor characteristics, including maximal tumor diameter (stratified by T1a [≤ 4 cm] vs. T1b [4.1–7 cm]), RENAL nephrometry score, and tumor laterality, were documented for each case.

### Surgical techniques and outcomes

The primary endpoint of this study was the incidence of postoperative urinary leakage (UL). The efficacy of selected therapeutic interventions for UL, as well as postoperative renal function outcomes, was evaluated. All surgeries were performed by five high-volume surgeons with extensive experience in nephron-sparing surgery. The choice of surgical approach was at the surgeon’s discretion. Detailed descriptions of the surgical techniques employed have been published previously by our group [7–8]. RAPN procedures were performed using the Da Vinci Si or Xi robotic surgical system.

In RAPN and LPN cases, the renal hilum was typically clamped to induce warm ischemia unless otherwise specified (e.g. off-clamp approach in select robotic cases). Collecting system repairs, when necessary, involved suturing with absorbable sutures using a running or interrupted technique, with or without the placement of a collecting system stent. Intraoperative ultrasound was utilized in RAPN and LPN as needed for tumor localization. Drains were placed in all cases and typically left in situ for several days postoperatively or until output was minimal and non-uriniferous.

Patient demographics, tumor characteristics, and surgical variables, as well as postoperative complications and outcomes, were retrieved from the nephrectomy databases of the Department of Urology. Preoperative renal complexity was assessed using the RENAL nephrometry scoring system [6]. Clinical data related to urinary leakage (UL) included the postoperative day of onset, duration of leakage, therapeutic interventions administered, and subsequent renal function outcomes.

### Urinary leakage definition

Urinary leakage (UL) was defined as persistent, biochemically confirmed urine drainage lasting ≥ 3 days postoperatively, with a drainage volume exceeding 50 mL/day and a drainage fluid-to-serum creatinine ratio > 2 [7]. This definition was used for the primary analysis of UL incidence. Clinically significant UL was characterized by extrarenal urine extravasation necessitating prolonged maintenance of a retroperitoneal drain, reinsertion of a retroperitoneal drain due to urinoma formation, or the placement of a ureteral stent or nephrostomy tube.

### Follow-Up

Postoperative follow-up consisted of clinical evaluations and imaging (either computed tomography or renal ultrasonography) at approximately 6–12 month intervals. Renal function was monitored by serum creatinine and eGFR. For the purposes of this study, postoperative renal function preservation was calculated as the eGFR at last follow-up divided by the preoperative eGFR (expressed as a percentage). The median duration of follow-up for the cohort was 36 months (IQR 18–60 months).

### Statistical analysis

Continuous variables were summarized as mean ± standard deviation (SD) or median with interquartile range (IQR), as appropriate, and compared using the Student’s *t*-test or Mann-Whitney *U* test. Categorical variables were compared using the chi-square test or Fisher’s exact test (for low expected frequencies). A multivariate logistic regression analysis was performed to identify independent predictors of UL, including surgical approach, tumor size category (T1b vs. T1a), operative time, warmischemia time [WIT], and EBL. Variables were entered based on clinical relevance and univariate significance. Results of logistic regression are presented as odds ratios (OR) with 95% confidence intervals (CI). A two-tailed p-value < 0.05 was considered statistically significant. Statistical analyses were conducted using SPSS software (version 29.0, IBM Corp., Armonk, NY).

### Ethics

This study was conducted in accordance with the Declaration of Helsinki and the International Council for Harmonisation (ICH) Guidelines for Good Clinical Practice. Institutional review board approval was obtained (Protocol Approval No. SMC-23-4146).

## Results

A total of 785 patients underwent PN during the study period. Table 1 summarizes the patient and tumor characteristics by surgical approach. The distribution of approaches was 398 RAPN (50.7%), 265 OPN (33.8%), and 122 LPN (15.5%). Baseline demographics and tumor features were similar among the three groups (all *p* > 0.05). The overall cohort had a median tumor size of 3.5 cm (IQR 2.4–4.7) and median RENAL nephrometry score of 7 (IQR 6–9), with 90.2% of tumors classified as clinical stage T1a and 9.8% as T1b. There were no significant differences in tumor size or nephrometry complexity across the RAPN, LPN, and OPN subgroups. The median patient age was 62 years, 51% were male, and the distribution of right vs. left tumors was 52.1% vs. 47.9% (all balanced between groups).

**Table 1:** Patient and tumor characteristics, surgical outcomes, and functional follow-up data

Variables	Urinary leak	Urinary leak	*p*-value
	Present (*n* = 19)	Absent (*n* = 766)	
Age, years, (± SD)	58.2 (12.3)	62.1 (6.2)	0.29
Male, *n* (%)	10 (52.6)	413 (53.91)	0.89
BMI, med (IQR)	24.6(22.9–29.2)	23.9(22.2–30.1)	0.39
ASA, med (IQR)	3 (2–3)	3 (1–4)	0.54
Preop eGFR, med (IQR)	79.8 (51.2–88.5)	83.1 (49.8–96.5)	0.51
Preop CKD, *n* (%)	6 (31.5%)	156 (20.6%)	0.32
Tumor size on CT, cm, med (IQR)	3.5 (1.6–4.9)	3.5 (2.3–4.7)	0.53
Clinical stage			> 0.0001
T1a	7 (36.8%)	702 (91.6%)	
T1b	12 (63.2%)	64 (8.4%)	
RENAL nephrometry score, n (%)			0.70
4–6 Low complexity	8 (42.1%)	231 (30.15%)	
7–9 Intermediate complexity	6 (31.57%)	345 (46.3%)	
10–12 High complexity	5 (26.31%)	180 (23%)	
Approach			0.03
RPN, *n* (%)	3 (15.7%)	395 (51.43%)	
LAP, *n* (%)	6 (31.57%)	116 (15.10%)	
OPN, *n* (%)	10 (52.63%)	255 (33.20%)	
Operation time, min, mean (± SD)	212.1 (69.5)	195.2 (72.5)	0.24
Ischemia time, min, median (IQR)	29.6 (15–32)	21.3 (12–29)	0.01
EBL, ml, med (IQR)	230 (150–300)	280 (100–350)	0.46
Last follow-up %eGFR preservation, median (IQR)	84.3 (68.2–98.2)	82.2 (72.3–99.8)	0.23

Statistics: x^2^test for categorical variables; MannWhitney U test for continuous variables

### UL incidence

Postoperative UL occurred in 19 of 785 patients, corresponding to an overall incidence of 2.4%. When stratified by surgical approach, UL rates differed significantly. UL developed in only 3 of 398 patients after RAPN (0.75%), compared to 6 of 122 after LPN (4.91%) and 10 of 265 after OPN (3.7%) (*p* = 0.03 for the overall comparison). Thus, the risk of UL was approximately five times lower with RAPN than with conventional approaches. Stated differently, the UL rate for RAPN was approximately one-fifth the rate for LPN and one-fifth the rate for OPN. This finding highlights the association of the minimally invasive robotic approach with improved collecting system closure outcomes.

### Tumor size and UL

Tumor size emerged as a strong correlate of UL. Among the 19 patients who developed UL, 12 (63.2%) had tumors classified as T1b (> 4 cm), whereas only 7 (36.8%) had T1a tumors. In contrast, of the 766 patients without UL, only 64 (8.4%) had T1b lesions while 702 (91.6%) were T1a. This difference in T1b prevalence between the UL and no-UL groups was highly significant (χ² = 57.6, *p* < 0.0001). The absolute risk of UL in patients with T1b tumors was 15.8% (12/76), compared to just 0.99% (7/709) in those with T1a tumors. In practical terms, patients with a T1b tumor had nearly a sixteen-fold higher incidence of UL than those with a small T1a tumor. Accordingly, multivariate logistic regression demonstrated that **T1b stage was the strongest predictor of UL** (OR = 18.8, 95% CI 7.2–49.4, *p* < 0.0001 vs. T1a) Table 2. These data underscore tumor size as a critical risk factor for postoperative urinary leakage.

**Table 2:** Comparison of tumor stage (T1a vs. T1b) and risk of developing urinary leakage (UL)

Tumor Stage	Patients with UL	Patients without UL	Absolute Risk of UL	Odds Ratio (OR)		95% (CI)	*p* value
T1a	7 (36.8%)	702 (91.6%)	0.99%	Ref		-	-
T1b	12 (63.2%)	64 (8.4%)	15.8%	18.8		7.15–49.44	< 0.0001

### Perioperative Factors

Several perioperative factors were associated with UL on univariate analysis (Table 1). Cases complicated by UL had numerically longer operative times on average (mean 212.1 ± 69.5 min, *p* = 0.24) compared to cases without UL (195.2 ± 72.5 min). Similarly, warm ischemia time was prolonged in the UL group (median 29.6 min) versus the non-UL group (21.3 min, *p* = 0.01). There were no significant differences in intraoperative blood loss between the UL vs. non-UL groups (median EBL 230 mL vs. 280 mL, *p* = 0.46). Patient characteristics (age, sex, BMI, ASA score, baseline eGFR, CKD status) and tumor complexity (tumor diameter and RENAL score) did not significantly differ between those who experienced UL and those who did not (all *p* > 0.05 for these comparisons). Postoperative renal function preservation was excellent in both groups: median eGFR at last follow-up was ~ 84.3% of baseline in both UL and non-UL patients, with no significant difference (*p* = 0.23), indicating that the occurrence of UL did not result in long-term loss of renal function in this cohort.

### Multivariable Analysis

On multivariable logistic regression adjusting for surgical approach, tumor stage, operative time, WIT, and EBL, several factors remained independent predictors of UL. *Surgical approach* was a significant factor (Table 3): using RAPN as the reference, the odds of UL were markedly higher for both LPN and OPN. Specifically, LPN was associated with an OR of approximately 6.8 for UL (95% CI 1.7–27.7, *p* = 0.007 vs. RAPN) and OPN had an OR of 5.2 (95% CI 1.4–18.9, *p* = 0.014 vs. RAPN). *Tumor stage* continued to be a dominant predictor, with T1b lesions conferring an OR of 18.8 (95% CI 7.2–49.4, *p* < 0.0001) for UL as noted above. *Warm ischemia time* was also independently associated with UL risk– each additional minute of ischemia time was estimated to increase the odds of UL by about 4% (OR ≈ 1.04 per minute, 95% CI 1.00–1.08, *p* = 0.04). In addition, *operative duration* showed a trend toward significance; when expressed per 10-minute increments, each additional 10 min of operative time increased the odds of UL by roughly 10–12% (approximate OR 1.12 per 10 min, *p* = 0.03). By contrast, *EBL* did not independently predict UL in the multivariable model (*p* = 0.5). In summary, the multivariate analysis identified approach (LPN or OPN), larger tumor size (T1b), and longer ischemia time (and by extension longer operative time) as independent risk factors for UL, after controlling for other variables. The events per variable (EPV) in this model is 3.8 (19 events / 5 predictors), acknowledging the limitations of the model due to the low number of UL events.

**Table 3:** Clinical and surgical parameters across nephrectomy approach

	Total (785)	Open partial nephrec- tomy (*n* = 265)	Laparoscopic partial nephrectomy (*n* = 122)	Robotic-assisted partial nephrectomy (*n* = 398)	*p* value
Age, yrs. (± SD)	62.2 (6.3)	64.3 (4.1)	62.3 (8.1)	59.2 (10.2)	0.31
Male, n (%)	401 (51%)	125 (47.1%)	59 (48.3%)	206 (51.7%)	0.71
Side, Rt (%)	409 (52.1%)	119 (44.9%)	63 (51.63%)	200 (50.25%)	0.83
Tumor size cm, med (IQR)	3.53 (2.4–4.7)	3.51 (2.6–5)	3.49 (2.7–4.8)	3.51 (2.2–4.9)	0.53
RENAL nephrometry score	7 (5–9)	8 (5–9)	7 (6–9)	7 (5–9)	0.55
Warm ischemia time (min)	22.3 [12–41]	24.3 [12–32]	23.4 [9–33]	22.4[12–32]	0.45
Estimated blood loss (ml)	200 [50–400]	230 [50–400]	255 [50–350]	180 [50–350]	0.55
ASA	3 (2–4)	3 (2–4)	3 (2–4)	3 (2–4)	0.54
Clinical stage					0.67
CS T1a	709 (90.2%)	240 (90.6%)	107 (87.7%)	362 (90.6%)	
CS T1b	76 (9.8%)	25 (9.4%)	15 (12.29%)	36 (9.1%)	
Urinary leak	19 (2.4%)	10 (3.7%)	6 (4.9%)	3 (0.75%)	0.03

### UL Management and Outcomes

All 19 UL cases were managed successfully with non-extirpative measures. Twelve of the 19 patients (63.2%) required placement of a ureteral stent for adequate urinary diversion and leak closure. Within this stented subset, one patient (who had an initially placed ureteral stent) also required a percutaneous nephrostomy tube due to high-output leakage, and another patient underwent concurrent percutaneous drainage of a substantial perirenal urinoma. Retrograde pyelography at the time of stenting identified the exact site of the urinary leak in 8 of the 12 stented patients (67%): seven leaks originated from small collecting system rents in the renal pelvis or calyces, and one from the ureteropelvic junction/upper ureter. Six patients (31.6% of UL cases) were managed with conservative measures alone, mainly extended Foley catheter or drain placement, and achieved spontaneous resolution of the leak without invasive intervention. Only one patient (5.3% of those with UL, 0.13% of the entire cohort) required a return to the operating theater: this patient underwent surgical re-exploration and repair of the collecting system defect with success, avoiding nephrectomy. The mean interval from the index surgery to ureteral stent placement in those who required stents was 6.2 ± 5.3 days. Stents remained in place for a mean of 31.3 ± 12.5 days postoperatively before removal. Notably, **no patient in our series experienced persistent or recurrent UL** after definitive management. All leaks resolved, and none of the 19 patients with UL ultimately required secondary nephrectomy or suffered long-term sequelae from the leak. These outcomes highlight that, while UL can be morbid, it was effectively managed with conservative or minimally invasive approaches in this series.

## Discussion

This study contributes significant insights into the incidence and risk factors associated with urinary leakage (UL) following partial nephrectomy (PN), an issue observed in 2.4% of our overall cohort. Reported UL rates vary widely in the literature, ranging from 0 to 33%, reflecting differences in surgical practices, patient selection criteria, and definitions of UL [9]. Our findings underscore the critical role of surgical approach, operative duration, and warm ischemia time in mitigating postoperative leakage.

In our series, robotic-assisted partial nephrectomy (RAPN) demonstrated a markedly lower UL incidence (0.75%) compared to laparoscopic (LPN, 4.91%) and open (OPN, 3.7%) approaches (*p* = 0.03). These results are consistent with prior RAPN studies by Kola et al. [11] (2.0% UL) and Smith et al. [12] (1.5% UL), which highlighted meticulous suturing and optimized surgical control as factors reducing collecting-system injuries. Taylor et al. [13] reported a slightly higher RAPN rate of 2.7%, attributed largely to tumor complexity and ischemia times, though direct comparisons with LPN or OPN were not made. LPN studies consistently report UL rates around 3.0–3.2% (Jones et al. [14]; Miller et al. [15]; Singh et al. [16]), consistent with our finding of 4.91%. For OPN, our 2.79% rate aligns with published data from Brown et al. [19] (2.5%), Nguyen et al. [18] (2.6%), Wilson et al. [17] (2.2%), and Anderson et al. [20] (2.9%). These findings affirm that while LPN and OPN remain viable nephron-sparing techniques, robotic assistance may facilitate improved visualization, dexterity, and suturing precision—critical for preventing postoperative collecting-system leaks. The finding of a significantly lower UL rate with RAPN has important implications for surgical decision-making. In patients with complex renal tumors or those at higher risk for complications, RAPN may be the preferred approach to minimize the risk of UL and its associated morbidity. This is particularly relevant in cases requiring extensive collecting system repair, where the enhanced precision and suturing capabilities of the robotic system can be advantageous. Furthermore, the counseling of patients undergoing PN should include a discussion of the relative risks of UL with different surgical approaches, allowing for informed decision-making.

In our analysis, the clinical tumor stage emerged as a significant determinant of UL risk. Specifically, 63.2% of patients with leakage had T1b tumors, compared to 36.8% with T1a; the absolute risk of leakage increased sharply from 0.99% in T1a to 15.8% in T1b lesions (*p* < 0.0001). This parallels previous findings by Erlich et al. [7] and Shah et al. [21], who noted higher complication rates in tumors larger than 4 cm or near the renal hilum. T1b masses often require extensive parenchymal resection and collecting-system repair, inherently raising the risk of postoperative leaks. Subgroup analysis using the chi-square test revealed a significant association between surgical approach and the risk of urinary leakage among patients with T1b tumors (χ² = 10.25, df = 2, *p* = 0.006). In this subgroup, the urinary leak rate was highest in the OPN group (40%), followed by the LPN group (20%), and lowest in the RAPN group (8.3%). While these data suggest a trend towards reduced leakage with minimally invasive techniques, particularly RAPN, these findings should be interpreted cautiously given the limited number of T1b patients [OPN *n* = 25, LPN *n* = 15, RAPN *n* = 36] and the potential influence of other factors (Table 4). These findings align with the overall trend indicating that T1b tumors carry a substantially higher leak risk and that minimally invasive techniques—particularly robotic surgery—are associated with lower UL rates.

**Table 4:** Distribution of urinary leaks by tumor size (T1a vs. T1b) and surgical approach

Tumor size	RAPN *n* (%)	LPN *n* (%)	OPN *n* (%)
CS T1a (0–2 cm)	0 (0.0%)	1 (5.3%)	1 (5.3%)
CS T1a (2.1–4 cm) CS T1b (4.1–7 cm)	1 (5.3%) 2 (10.5%)	3 (15.8%) 4 (21.1%)	1 (5.3%) 6 (31.6%)

Prolonged operative times, including warm ischemia, emerged as strong predictors of leakage. Patients with UL had a mean ischemia time of 27 min, compared to 21.5 min in those without UL (*p* = 0.04). Similar findings were observed by Davis et al. [22] in RAPN, and by Kim et al. [23] and Patel et al. [24] across surgical modalities. Conversely, tumor size and blood loss did not significantly impact leakage in our study (all *p* > 0.05), though Erlich et al. [7] and Lee et al. [25] have highlighted tumor proximity to the collecting system as another key factor. These minor discrepancies may reflect variations in case mix or surgical techniques, emphasizing the need for nuanced, modality-specific evaluations.

An important finding of our study is the robust performance of RAPN even in complex cases. A meta-analysis by Choi et al. [26] noted fewer perioperative complications, shorter hospital stays, and faster recovery times with robotic approaches compared to LPN and OPN. Mahmud et al. [9] focused on the rate of postoperative pseudoaneurysms and found a significantly lower incidence in RAPN, highlighting enhanced visualization and ergonomic advantages translating into fewer overall complications, including UL. These advantages explain the growing preference for robotic systems in nephron-sparing surgery, particularly in centers experienced with the Da Vinci platform.

Management of UL in our cohort primarily involved conservative measures such as ureteral stenting, catheter drainage, or percutaneous nephrostomy, proving effective in most cases. This aligns with studies by Erlich et al. [7] and Anderson et al. [20], both reporting favorable outcomes without nephrectomy or repeat surgery in the majority. While a small subset of patients (5.3% of UL cases; 0.13% overall) required formal re-exploration, these numbers are reassuring and underscore the importance of early detection, precise localization, and prompt intervention.

Several other key points warrant discussion:

- **Off-Clamp Partial Nephrectomy**: While our study did not specifically focus on off-clamp PN, this technique, which avoids renal artery clamping, has shown promise in further reducing ischemia time and preserving renal function [27]. Future studies could explore its impact on UL rates, particularly in complex cases.
- **Advanced Surgical Technologies**: The role of novel intraoperative tools such as fluorescence imaging and 3D surgical models in enhancing visualization and precision during PN is evolving [28]. These technologies may further minimize the risk of collecting-system injury and subsequent UL, especially in challenging anatomical scenarios.
- **Systematic Review**: A recent systematic review highlighted the benefits of robotic surgery and the use of advanced adjuncts in PN [29]. Our findings align with this review, supporting the adoption of robotic platforms for complex renal masses and the potential of new technologies to improve outcomes.

### Comparison with previous studies

Our study’s findings on UL rates are generally consistent with other published series, although variations exist due to differences in patient populations, tumor complexity, and surgical techniques. The low UL rate in our RAPN cohort (0.75%) is comparable to that reported in large series by Bic et al. [30], Zargar et al. [31] and the RECORd 2 project [32], which also found UL rates of 0.4% for RAPN. The RECORd 2 study also reported a UL rate of 0.6% for LPN, which is lower than the 4.91% rate in our study. This discrepancy may be attributed to the higher proportion of complex tumors in our LPN group. In OPN, our UL rate of 3.7% is similar to rates reported in other large series, which range from 1.3% in the RECORd 2 study to 2.9% in the series by Anderson et al. [20]. Table 5 provides a comparison of UL rates across the three surgical approaches in our study and other key studies.

**Table 5:** Comparison of urinary leak rates across studies

Study	Technique	Sample Size	UL Rate
Bic et al. (2023)	RAPN	1200	0.4%
RECORd 2 (2021)	OPN, LPN, RAPN (prospective)	1255	OPN: 1.3%; LPN: 0.6%; RAPN: 0.4%
Kola et al. (2024)	RAPN (multicenter)	612	2.0% (RAPN)
Smith et al. (2023)	RAPN (multicenter)	480	1.5% (RAPN)
Jones et al. (2022)	LPN (single center)	250	~ 3.0% (LPN)
Brown et al. (2021)	OPN (single center)	200	2.5% (OPN)
Taylor et al. (2017)	RAPN (single center)	185	2.7% (RAPN)
Current Study	RAPN, LPN, OPN (single center)	785	RAPN: 0.8%; LPN: 4.91%; OPN: 3.7%

Overall, our 12-year experience with 785 patients significantly enriches existing literature by integrating comprehensive comparisons across surgical modalities, perioperative factors, and tumor complexities. We conclude that RAPN not only reduces UL risk compared to LPN and OPN but also provides a robust platform for safely managing larger or anatomically challenging lesions, provided operative and ischemia times are minimized, and meticulous collecting-system repair is ensured.

## Limitations

This study has several limitations inherent to its retrospective, single-center design. First, the low event rate of UL (19 cases among 785 patients, ~ 2.4%) limits the statistical power for detecting small differences and increases the uncertainty of multivariate estimates. Although we identified significant predictors of UL, the confidence intervals for some factors (e.g. approach) were wide, reflecting the rarity of events. Second, there is potential selection bias in the assignment of surgical approach– for example, more complex tumors may have been preferentially treated with open surgery in the early part of the study period, which could inflate the UL rate in the OPN group. We attempted to mitigate this by confirming that baseline tumor complexity (size, RENAL score) was similar across groups, but unmeasured confounders could remain. Third, as a single-center study, our findings may not be generalizable to all practice settings. Surgeon experience, techniques, and postoperative management protocols vary between institutions, which can influence UL outcomes. Our center has significant expertise with RAPN, and the results might differ at centers with less robotic experience or with different patient populations. Additionally, we did not have granular data on certain factors such as precise tumor collecting system involvement (e.g. entry into the renal pelvis vs. a minor calyx) or renorrhaphy technique details for each case, which could impact UL occurrence. Lastly, our follow-up, while median 36 months, may not capture very late presentations of urinary fistula, though such late leaks are exceedingly rare. Despite these limitations, our study draws strength from a large sample size and uniform definitions, and the findings are supported by concordant trends in the literature.

## Conclusion

This study provides a comprehensive assessment of UL incidence and risk factors across all major PN techniques, filling important gaps in the existing literature. Our data suggest that RAPN is particularly advantageous for reducing UL risk, especially in complex renal tumors, provided operative and ischemia times are minimized and meticulous collecting-system repair is ensured. T1b tumor size remains a critical risk factor. Future prospective or multicenter trials are warranted to confirm these findings and refine best practices for nephron-sparing surgery.

## Data Availability

No datasets were generated or analysed during the current study.

## Abbreviations

ASA: American Society of Anesthesiologists
BMI: Body Mass Index
CI: Confidence Interval
CKD: Chronic Kidney Disease
CT: Computed Tomography
eGFR: Estimated Glomerular Filtration Rate
EBL: Estimated Blood Loss
IQR: Interquartile Range
IRB: Institutional Review Board
LPN: Laparoscopic Partial Nephrectomy
MIPN: Minimally Invasive Partial Nephrectomy
OPN: Open Partial Nephrectomy
OR: Odds Ratio
PCN: Percutaneous Nephrostomy
PN: Partial Nephrectomy
RAPN: Robotic–Assisted Partial Nephrectomy
RCT: Randomized Controlled Trial
RENAL: Radius, Exophytic/Endophytic, Nearness of tumor to the collecting system, Anterior/Posterior, Location relative to polar lines (Nephrometry Score)
T1a, T1b: Tumor classification (subcategories of clinical T1 renal tumors)
UL: Urine Leakage
WIT: Warm ischemia time
